# Mother and Infant Nutrition Investigation in New Zealand (MINI Project): Protocol for an Observational Longitudinal Cohort Study

**DOI:** 10.2196/18560

**Published:** 2020-08-27

**Authors:** Ying Jin, Jane Coad, Shao J Zhou, Sheila Skeaff, Cheryl Benn, Nicholas Kim, Rachael L Pond, Louise Brough

**Affiliations:** 1 School of Health Sciences College of Health Massey University Palmerston North New Zealand; 2 School of Food and Advanced Technology College of Sciences Massey University Palmerston North New Zealand; 3 School of Agriculture, Food and Wine and Robinson Research Institute Faculty of Sciences University of Adelaide Adelaide Australia; 4 Department of Human Nutrition University of Otago Dunedin New Zealand; 5 MidCentral District Health Board Palmerston North New Zealand; 6 School of Health Sciences College of Health Massey University Wellington New Zealand; 7 Institute of Education College of Humanities and Social Sciences Massey University Palmerston North New Zealand

**Keywords:** iodine, selenium, iron, thyroid hormone, breastfeeding women, postnatal depression, infant neurodevelopment, thyroid, maternal health, nutrition

## Abstract

**Background:**

Thyroid dysfunction is associated with cognitive impairment, mood disturbance, and postnatal depression. Sufficient thyroid hormone synthesis requires adequate intake of iodine, selenium, and iron. Iodine deficiency was historically a problem for New Zealand, and initiatives were introduced to overcome the problem: (1) mandatory fortification of all bread (except organic) with iodized salt (2009) and (2) provision of subsidized iodine supplements for pregnant and breastfeeding women (2010). Subsequent to these initiatives, most adults and children have adequate iodine status; however, status among breastfeeding women and their infants remains unclear. This paper outlines the methodology of the Mother and Infant Nutrition Investigation (MINI) study: an observational longitudinal cohort study of breastfeeding women and their infants.

**Objective:**

This study will determine (1) women’s iodine intake and status among supplement users and nonusers; (2) women’s intake and status of iodine, selenium, and iron relating to thyroid function; (3) associations between women’s selenium status, thyroid function, and postnatal depression; (4) infants’ iodine and selenium status relating to first year neurodevelopment.

**Methods:**

Breastfeeding women aged over 16 years with a healthy term singleton infant were recruited from Manawatu, New Zealand. Participants attended study visits 3, 6, and 12 months postpartum. Maternal questionnaires investigated supplement use before and after birth, iodine knowledge, and demographic information. Dietary assessment and urine, blood, and breast milk samples were taken to measure iodine, selenium, and iron intake/status. The Edinburgh Postnatal Depression Scale was used repeatedly to screen for postnatal depression. Thyroid hormones (free triiodothyronine, free thyroxine, thyroid stimulating hormone, thyroglobulin, antithyroglobulin antibodies, and antithyroid peroxidase) were measured in blood samples, and thyroid gland volume was measured by ultrasound at 6 months postpartum. Infant iodine and selenium concentrations were determined in urine. The Ages and Stages Questionnaire was used to assess infant development at 4, 8, and 12 months.

**Results:**

Data collection was completed. Biological samples analysis, excluding nail clippings, is complete. Data analysis and presentation of the results will be available after 2020.

**Conclusions:**

This study will provide data on the current iodine status of breastfeeding women. It will also provide a greater understanding of the three essential minerals required for optimal thyroid function among breastfeeding women. The prospective longitudinal design allows opportunities to examine women’s mental health and infant neurodevelopment throughout the first year, a crucial time for both mothers and their infants.

**Trial Registration:**

Australian New Zealand Clinical Trials Registry ACTRN12615001028594; https://www.anzctr.org.au/Trial/Registration/TrialReview.aspx?id=369324

**International Registered Report Identifier (IRRID):**

DERR1-10.2196/18560

## Introduction

### Background

Postpartum women experience abnormalities in thyroid function at twice the prevalence of the general population [1]. Thyroid hormone is essential in maintaining the human body’s metabolism, temperature (thermoregulation), and psychological mood [2]. In a developing brain, thyroid hormone is responsible for adequate myelination, neuron cell maturation, and central nervous system development [3]. Optimal thyroid function relies on adequate biosynthesis of thyroid hormones, which depends on three dietary minerals: iodine, selenium, and iron [4,5]. Pregnancy increases thyroid hormone turnover; thus, women with limited thyroidal reserve or marginal iodine deficiency are at increased risk to develop thyroid dysfunction after birth [6]. This is one of the most common endocrine disorders that postpartum women experience [7].

Iodine is the major component of thyroid hormones, but it is also a regulator for the synthesis and secretion of the thyroid hormones triiodothyronine (T3) and thyroxine (T4). Selenium, as a component of the selenocysteine-containing proteins glutathione peroxidase, protects the thyroid gland from oxidative damage [5]. Selenoproteins are required to convert T4 to T3, the active form of thyroid hormone. Iron is required for heme-dependent thyroperoxidase activity, which is required for the synthesis of adequate thyroid hormone. Selenium deficiency and iron deficiency anemia may negatively affect thyroid hormone synthesis by impairing selenium- and iron-dependent enzyme activities, even if iodine status is adequate [5]. Most previous research has investigated iodine, selenium, and iron intake/status separately or a combination of any two of them among women of childbearing age [8] and postmenopausal women [9]. However, further research is needed to explore all three micronutrients together, acknowledging their close relationship in thyroid hormone synthesis.

Thyroid dysfunction is a significant health issue in New Zealand, with women diagnosed at 5 times the prevalence in men [10,11]. Concerning adequate thyroid function, iodine, selenium, and iron play important roles. In New Zealand, soils provide low levels of available iodine and selenium, resulting in low concentrations in the food supply [12], hence in the diet [13]. Iodine deficiency in early life is associated with impaired neurodevelopment [14]. Iodine deficiency was a concern in New Zealand in the early years of the 20th century, but its prevalence was mostly reduced through the introduction of iodized salt in the 1930s. However, since the 1990s, a number of studies in New Zealand have shown iodine deficiency has reemerged in adults [15], pregnant and breastfeeding women [16,17], school children [18] and breastfed infants and toddlers [19]. To improve iodine status in New Zealand, two government initiatives were introduced: mandatory iodized salt in commercially made bread products from September 2009 and the provision of iodine supplementation for all pregnant and lactating women in 2010 [20]. Although recent studies suggested that adults [21,22] and children [23] in New Zealand may now have adequate iodine intake/status, both pregnant and breastfeeding women remain deficient. A pilot study of a small sample of self-selected highly educated pregnant and breastfeeding women assessed urinary iodine excretion, breast milk iodine concentration, and blood thyroglobulin and suggested iodine deficiency [24]. There is a need for a more robust investigation into the iodine status of postpartum women and their infants from a wide range of socioeconomic backgrounds.

Low selenium status in New Zealand has been partially reversed by increased consumption of imported flour from Australia (which generally has higher selenium concentrations than flour produced in New Zealand) [25,26]. In addition, both pregnant and breastfeeding women have an increased requirement for dietary selenium due to the demands from the fetus and breastfed infants. Previous research, which investigated selenium status among postpartum women and their infants in New Zealand 20 years ago [27], measured urinary selenium excretion and plasma selenium and indicated that such women were at risk of selenium deficiency. To our knowledge only one small study of breastfeeding women, by our research group, assessed dietary selenium, urinary selenium excretion, and breast milk selenium concentration and suggested selenium inadequacy was still a concern [28]. There remains a lack of research investigating selenium status among postpartum women and their infants.

Health professionals closely monitor the iron status of women during pregnancy. However, after birth, management of iron status can be inconsistent. Results from a UK multicenter study reported only 50% of postpartum women had hemoglobin levels checked after delivery (with 30% of those women confirmed as anemic), while the overall iron stores of participating women remained unexamined [29]. Generally, postpartum women’s iron status recovers as a consequence of cessation of menstrual bleeding since conception or a minimal secretion of iron via breast milk if breastfeeding [30]. However, if women have suffered iron deficiency before and/or during pregnancy and/or have experienced significant blood loss during the birth, their iron status may not reach optimal levels even if an intervention subsequently occurs. A New Zealand study of 186 women found 77% of women were not tested for hemoglobin levels after birth. Further, out of those most at risk (with low iron status during late pregnancy and high blood loss, exceeding 500 mL, during birth), few women were then retested for their iron status after 10 days postpartum [31]. Iron status of postpartum women remains largely underreported.

Moreover, low serum selenium has been identified as an independent risk factor for depression, [32] and selenium supplementation has been observed to reduce postnatal depression [33]. Postnatal depression is one of the main disorders women experience postnatally, its onset being timed at 6 weeks to 6 months after birth. Most women will recover from postpartum depression, though approximately one-quarter of affected women report being depressed when their infant reaches their first birthday [34]. Using the measured criteria of postnatal depression on the Edinburgh Postnatal Depression Scale (EPDS), the prevalence of postnatal depression in New Zealand was about 8% in 1994 and 16% in 2006. In the 2015 New Mothers’ Mental Health Survey [35], the prevalence was 14% and is now recorded as the most common disorder for mothers in their first year after childbirth [36].

Of additional concern, mothers are often reluctant or unable to seek help when they experience symptoms of postnatal depression [35]. Such underdiagnosed and untreated mental health conditions affect both the mother and their children’s ongoing cognitive, emotional, and behavioral development [37]. Despite other social and psychological etiology of depression, potential links between micronutrient status, thyroid hormone, and the risk of postpartum depression need to be further explored. This may help develop new preventive approaches to lowering the risks of postpartum depression.

### Study Objectives

The study’s primary outcomes include (1) investigating breastfeeding women’s iodine intake and status among supplement users and nonusers following the implementation of two government initiatives to improve iodine status; (2) examining maternal iodine, selenium, and iron intake status; and (3) exploring iodine, selenium, and iron status in maternal thyroid function.

In addition, the study provides preliminary data on possible associations between women’s selenium status, thyroid function, and postnatal depression over a 1-year period and infants’ iodine and selenium status in relation to neurodevelopment during their first year of life. Ultimately, this research will inform a future larger study of potential variables impacting maternal thyroid function and the risk of postnatal depression, together with early infant neurodevelopment.

## Methods

### Study Design and Overview

The Mother and Infant Nutrition Investigation (MINI) study is an observational longitudinal cohort study spanning the first year postpartum. It was approved by the Health and Disability Ethics Committee (15/NTA/172) in December 2015. The study’s ethics approval was registered with the Royal New Zealand Plunket Ethics Committee in June 2016. The MidCentral District Health Board in New Zealand also approved the study. The study was registered with the Australian New Zealand Clinical Trials Registry [ACTRN12615001028594].

The study is being conducted in the Human Nutrition Research Unit at Massey University, Palmerston North, New Zealand. The first study visit for participants is at approximately 3 months postpartum, and follow-up assessments take place at 6 months and 12 months postpartum (Multimedia Appendix 1).

### Selection Criteria

The target population for the study was healthy breastfeeding women aged over 16 years who had birthed a healthy term singleton infant 3 months prior. Women were excluded if they developed significant health problems, such as metabolic disease or cancer. Women were excluded if they had been diagnosed or treated at any time for hyperthyroidism or hypothyroidism. Participants were required to live within or near the local Palmerston North area and be able to attend Massey University for scheduled study visits. Women of any ethnic and socioeconomic status were eligible.

### Recruitment and Participation

Posters to promote the study were placed at selected sites (general practitioner surgeries, midwifery clinics, pharmacies, antenatal classes, ultrasound clinics, maternal wards in hospitals, local community playgroups, and early childhood centers, etc). Local newspapers and social media sites were used to publicize the study. Local midwives, childbirth educators, and lactation consultants were asked to raise awareness of the MINI study to their clients. An effort was made to recruit women from a wide range of socioeconomic backgrounds and ethnic groups, including Maori, Pacific Islanders, and Asian women. Potential participants responded by recording an expression of interest online or via telephone or email. Prospective participants were provided with a study information sheet. Interested participants then completed a screening questionnaire to ensure eligibility. Written informed consent was obtained from all participants before their enrollment in the study. Mothers also gave written consent to their infants’ participation in the study. After providing informed consent, participants were assigned a unique identifier code and scheduled for their first study visit.

### Sample Size Calculation

The main outcome measure was iodine excreted per day, and the sample size was calculated using G*Power 3.1 (Heinrich Heine University) based on data (mean and standard deviation) from a preliminary study of breastfeeding women [24]. Calculation used 1-way analysis of variance with two groups (95% power, 𝛼=.05, 2-tailed) and three repeat measures; 80 participants were needed, using expected mean daily urine iodine concentrations of 140 and 100 µg/d for iodine supplement users and nonusers, respectively, and a standard deviation of 60.

### Outcome Measures

#### Questionnaires

At the initial visit, general baseline questions were asked about salt and supplements use, nutrition knowledge of iodine, tobacco and alcohol use, breastfeeding patterns, general health, and demographic information (including age, ethnicity, educational attainment, household size, and income). Potential changeable information including tobacco and alcohol use, breastfeeding patterns, and general health was also sought at the second and third visits.

Participants were assessed about their general health and that of their infants by online questionnaire when infants reached 6 months and 12 months of age. During the postpartum period, stress may negatively affect immunity, and the occurrence of infection symptoms can be an estimated measurement of postpartum immune function. The Carr Infection Symptom Checklist, which has been validated for use with postpartum women [38], was used to measure the symptoms of infection experienced by the mother since the birth. The Infant Symptom Checklist (which reports the frequency of symptoms of common illnesses in young infants) was used to measure the health of infants [38].

The 10-item EPDS was completed online by participating women to assess any symptoms of depression and anxiety over the previous 7 days. Women recorded severity of symptoms on a 4-point scale [39]. Specified anxiety disorders were evaluated using the EPDS-3A, a cluster of selected question items numbered 3, 4, and 5 from the original EPDS [40]. This is a validated tool to screen for probable anxiety and depression during the postpartum period. A cutoff point of 13 or above was used to define high levels of depressive symptoms [35]. Any woman whose score equaled 13 or above was advised to see her general practitioner for further evaluation as well as being provided with an information sheet containing postnatal depression services in New Zealand. Only study participants with the correct link supplied via emails could complete these questionnaires. All questions were answered in the same order. Participants could not go back to change their answers once the questionnaire was completed. Answers from incomplete questionnaires may be used for analysis.

The first year of infant neurodevelopment was assessed using a parent-completed Ages and Stages Questionnaire (ASQ) when the infant was aged 4, 8, and 12 months [41]. These questionnaires were self-administered and completed in hard copies. This screening tool uses parent observation to assess child development and behavior and records results in 5 developmental domains: communication, gross motor, fine motor, problem solving, and personal-social. There are 6 questions in each domain, with answers of yes, sometimes, or not yet. A yes indicates reaching the achievement with 10 points awarded, a sometimes indicates partial achievement with 5 points awarded, and a not yet indicates not achieved with 0 points awarded. The sum score of each domain was calculated and compared with the cutoff scores reached, which were derived empirically by subtracting 2 standard deviations from the mean for each area of development [41]. A score below the cutoff point indicates a fail on the ASQ. The questionnaires were used to assess the relationship between maternal and infant iodine and selenium status, maternal iron status, and recorded early child neurodevelopment.

#### Dietary Intake

To assess participant dietary intake including nutrients that may be associated with mental health and child development including iodine, selenium, and iron intake, participants were asked to complete a weighed 4-day diet diary within 2 weeks of the initial study visit. All 4 days were consecutive and included one weekend day. Each participant was requested to record food items, brands, amount consumed, and the content of the nutritional information panel if applicable. All food and beverage items consumed were weighed and measured with a QM-7288 electronic kitchen scale (Digitech), and household measurement cups and spoons were provided. The Digitech scale can weigh up to 5 kilograms with an accuracy to 1 gram; all women were shown how to use the scale to quantify food items. All participants received both written and oral instructions on how to complete the record, which included a written example of a 1-day food record. Women were also asked to include dietary supplements consumed. When eating or dining out, participants were asked to estimate the portion size of all food eaten. The food record and equipment were collected or return posted 2 weeks after the initial visit.

A 69-item self-administrated semiquantitative iodine- and selenium-specific food frequency questionnaire, adapted from an Australian study of pregnant women [42], was used to estimate habitual maternal iodine and selenium intake at the first and third study visits. An iron-specific food frequency questionnaire, validated by other female population groups in New Zealand, was used to assess women’s iron-related dietary patterns [43] at the second study visit. Within 2 weeks of this visit, participants also completed a 3-day estimated food dietary record for their infants to enable assessment of infant nutrient intakes at weaning periods.

All dietary data were entered into Foodworks 9 Professional (Xyris Pty Ltd) online and analyzed using data sets from the New Zealand Foodfiles 2016 to estimate nutrient intake. When food items were not included in Foodfiles 2016, new food items were created based on the information directly provided by participants (ie, food packages) or from appropriate international databases from Australia and the United States. Estimates for iodine concentrations of categories of bread (eg, white, fiber white, fruited, mixed grain) were based on data from the Ministry of Primary Industries [22], since iodine content has not been determined for all commercially made bread in New Zealand after the mandatory fortification of bread with iodized salt. It was difficult to quantify the amount of discretionary salt added to food. However, for women who reported using iodized salt, 48 µg of iodine (equivalent to 1 g of salt) was added to their iodine intakes [21]. Dietary supplements used by participants were entered into Foodworks as a new food item based on nutritional information obtained from the manufacturers. To ensure accuracy and completeness, a registered nutritionist (YJ) checked all dietary data and then transferred the data to SPSS Statistics (IBM Corporation) version 23 for statistical analysis.

#### Anthropometry

Maternal and infant anthropometry measurements were obtained at each study visit. Women’s weight was measured using the same annually calibrated weighing scale with a capacity of 150 kilograms (Detecto). Before standing on the scale, participants were asked to remove their shoes and to wear minimum clothes. Body weight was recorded to the nearest 0.1 kilogram. Height was measured by using a Toledo stadiometer and recorded to the nearest millimeter [44]. Maternal body composition was determined using both bioelectrical impedance analysis (InBody230, InBody Co) and air displacement plethysmography (BodPod, COSMED SRL). Measurements were completed under the following conditions: minimal clothing, wearing swimming cap, before midday, after urination, normal room temperature (20℃ to 25℃), with no exercise, eating, drinking, or bathing/showering within 2 hours prior to measurement (preferably completing the measurement after breastfeeding the baby). On the day of the test, quality control steps for BodPod were carried out by following the manufacturer’s instructions, with acceptance criteria being volume ±100 mL of actual volume and standard deviation ≤75 mL.

Infant recumbent length was measured crown to heel using an infant length board and recorded to the nearest millimeter. Infant weight (without clothing and diapers) was measured using a baby weighing scale (Nagata Scale Co Ltd) and recorded to the nearest 10 grams. Infant head circumference was measured over the most prominent part on the back of the head (occiput) and just above the eyebrows (supraorbital ridges) by using a flexible, nonstretch tape [44] and recorded to the nearest even millimeter.

#### Ultrasound Measurement of Thyroid Gland Volume

A portable ultrasound (uSmart 3200T Ultrasound System, Teratech Corp) equipped with a linear transducer (7 to 15 mHz) was used for the thyroid measurement. Women were examined in a supine position (an adequate neck extension was achieved by placing pillows under the shoulders). Longitudinal and transverse scans were performed. Measurements of anteroposterior diameter and width (mediolateral diameter) were obtained with electronic calipers on a transverse image. The maximum lobe length was measured on a longitudinal width. The total volume of each thyroid gland was the sum of the volumes of left and right lobes, excluding the volume of the isthmus but including any nodules and/or cystic areas. The formula used to calculate the volume for each lobe is anteroposterior diameter × width × length × 0.479 [45]. A total volume greater than 18 mL was defined as thyroid enlargement based on the normative thyroid volume in iodine sufficient populations [46]. Any participant with observed abnormalities was referred to clinical health professionals for further assessment.

#### Biomarker Analysis

During each study visit, spot urine samples from each participating woman and her infant were collected to assess iodine, selenium, and creatinine excretion. All maternal spot urine samples were collected in the morning and immediately frozen and stored at –20℃. Infant urine was collected using a 100 mL pediatric urine bag placed inside the diaper and checked every 10 minutes. The collected urine was frozen and stored at –20℃ for later analysis. Spot urine samples can be used to estimate iodine status of a population but not for individual iodine deficiency diagnosis [47]. It was not possible to estimate dietary iodine and selenium intake for lactating women via urine as we were unable to determine the daily loss of selenium from breast milk. As creatinine output is relatively constant, the adjusted iodine/creatinine ratio (µg iodine per g creatinine) can be used as a proxy measure of iodine excretion [48].

Lactating women were asked to provide a breast milk sample (approximately 30 to 50 mL) at each visit using an Allegro electric breast pump (Unimom NZ) if required. All breast milk samples were collected before noon on the study visit day, and timing of breast milk collection was not standardized. Breast milk samples were analyzed for iodine and selenium concentration, allowing for estimations of infant intake of iodine and selenium based on 750 mL/d of milk production [49].

Iodine and selenium concentration in both urine and breast milk samples were determined by an accredited commercial laboratory (Hill Laboratories) using inductively coupled plasma mass spectrometry (ICP-MS) [50]. Quality control procedures included analysis of blanks, analytical repeats, and certified reference material to ensure accuracy and precision. The Massey University Nutrition Laboratory measured creatinine using the Jaffe method in a Flexor E (Vital Scientific) biochemistry analyzer.

To assess further selenium status, toenail clippings from women and nail clippings from infants were collected. Toenail clippings have been used to determine selenium concentrations in large cohort or epidemiological studies, such as for the preeclampsia risk in pregnant women [51]. The instruction for sample collection was explained to participants during each study visit and nail clippings were self-collected by participating women at home, with the collected samples brought back by the participants at the following study visit. All toenail clippings were stored at room temperature prior to analysis. Nail clipping samples will be prepared by using the method adapted from nail zinc analysis [52]. This involves washing all nail clipping samples by using 5 minutes contact with 25 mL portions in the order of acetone, water, acetone, water, and water [53]. Selenium concentration will be measured by ICP-MS.

During the second study visit, to assess blood hemoglobin concentrations, the handheld HemoCue Hb 201+ device (HemoCue America) was used, a standard in hemoglobin point-of-care testing [35,54]. It requires a finger prick and wicking of capillary blood into a pretreated microcuvette for analysis. Quality tests using external, liquid controls were necessary for each day of instrument use prior to sample analysis.

A qualified and experienced phlebotomist collected nonfasting maternal venous blood samples (22 mL) at the second study visit. Samples were centrifuged and aliquoted into microcentrifuge tubes prelabeled with participant unique sample ID and then stored at –80℃. In conjunction with the hemoglobin results, collected maternal venous blood samples were used to determine iron status by measuring soluble transferrin receptors and serum ferritin (using the chemiluminescent microparticle immunoassay [CMIA] method), which reflects iron storage, but if serum ferritin levels are increased during infection or inflammation, it may mask any iron deficiency results [55]. Therefore, an inflammatory marker, C-reactive protein, was measured (tested by an immunoturbidometric method analyzed on an Abbott C Series analyzer [Abbott Labs]).

Venous blood samples were assayed for hormonal biomarkers: free T3, free T4, and thyroid stimulating hormone via CMIA method; thyroglobulin (Tg, Beckman Coulter Access method); and antithyroglobulin antibodies (anti-Tg, CMIA method) at Canterbury Health Laboratories. Serum thyroglobulin has been suggested as an alternative method to assess individual iodine status reflecting a period of months [56]; to avoid potential underestimation of thyroglobulin, anti-Tg and antithyroid peroxidase (anti-TPO) were measured. Selenium status was assessed by determining the biomarker plasma selenium via ICP-MS method [57].

Details of data and biological samples collected from both mothers and infants throughout the study period are summarized in Tables 1 and 2.

**Table 1:** Summary of outcome measures collected from participating women and their infants.

Outcome		Visit 1	Visit 2	Visit 3
**Dietary intake**				
	Maternal 4-day dietary diary	x		
	Maternal food frequency questionnaire–iodine/selenium	x		x
	Maternal food frequency questionnaire–iron		x	
	Infant 3-day dietary diary		x	
**Anthropometry**				
	Maternal weight and height	x	x	x
	Maternal body composition via BodPod and BIA^a^	x	x	x
	Infant weight, height, and head circumference	x	x	x
**Biochemistry**				
	Maternal spot urine samples	x	x	x
	Maternal breast milk samples	x	x	x
	Maternal toenail clipping samples	x	x	x
	Maternal venous blood samples		x	
	Maternal capillary blood samples		x	
	Infant spot urine samples	x	x	x
	Infant nail clipping samples	x	x	x
**Others**				
	Maternal thyroid gland volume via ultrasound		x	
	Maternal Edinburgh Postnatal Depression Scale results	x	x	x
	Maternal self-reported health questionnaire	x	x	x
	Maternal iodine nutritional knowledge questionnaire	x		
	Infant health questionnaire reported by mothers	x	x	x

^a^BIA: bioelectrical impedance analysis.

**Table 2:** Analysis from biological data collected at each study visit for the Mother and Infant Nutrition Investigation study cohort.

Samples		Visit 1			Visit 2			Visit 3	
		Mothers	Infants	Mothers		Infants	Mothers		Infants
**Spot urine**									
	Iodine	x	x	x		x	x		x
	Selenium	x	x	x		x	x		x
	Creatinine	x		x			x		
**Breast milk (if available)**									
	Iodine	x		x			x		
	Selenium	x		x			x		
**Blood**									
	Iodine status^a^			x					
	Selenium status^b^			x					
	Iron status^c^			x					
	Thyroid function^d^			x					
**Nail clippings for selenium**									
	Toenails	x	x	x		x	x		x
	Fingernails		x			x			x

^a^Iodine status: testing thyroglobulin and antithyroglobulin.

^b^Selenium status: testing plasma selenium.

^c^Iron status: testing hemoglobin, serum ferritin, soluble transferrin receptors, and C-reactive protein.

^d^Thyroid function: testing serum free triiodothyronine, free thyroxine, thyroid stimulating hormone, and antithyroid peroxidase.

### Statistical Analysis

Statistical analysis will be performed using SPSS Statistics version 23. The Shapiro-Wilk test will be used to test for data normality. Nonparametric data will be expressed as median (25th, 75th percentile), and parametric data will be expressed as mean and standard deviation. Bivariate correlations will be tested using the nonparametric Spearman 𝜌 correlation coefficient. Repeated-measures analysis of variance will be used to calculate continuous variables between groups. Nonparametric Mann-Whitney *U* test (2-tailed) will be used to examine iodine intake and status between supplement users and nonusers. Multiple regression models analysis will be used to determine the associations between iodine, selenium, iron status, and thyroid function, as well as considering confounding factors. Multivariate analysis will be used to examine possible associations between women’s selenium status, thyroid function, and postnatal depression and infant first year neurodevelopment.

## Results

**Figure 1:**
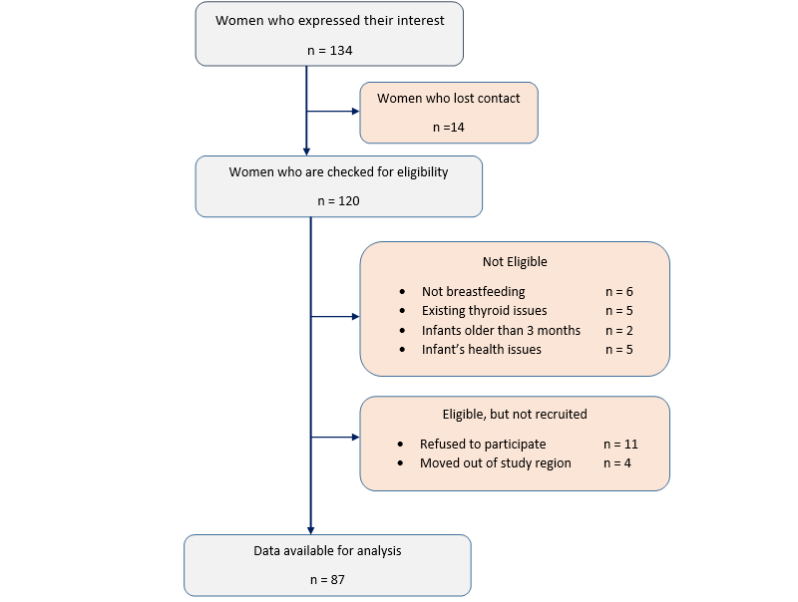
Flow chart showing recruitment for the Mother and Infant Nutrition Investigation study.

Recruitment traversed the 19-month period between June 2016 and December 2017, and a sample of 91 women-infant pairs was enrolled (Figure 1). Data collection has been completed. Biological samples analysis, excluding nail clippings, is complete. Data analysis and presentation of the results will be available after 2020.

## Discussion

### Summary

A unique aspect of this study is that it will investigate all three micronutrients responsible for adequate thyroid hormone synthesis concurrently, rather than each separately in isolation. This observational longitudinal cohort study will measure the iodine and selenium status of women repeatedly in their first year after birth, which provides an evaluation of their nutritional status. Iodine status among supplement users and nonusers will provide up-to-date data on this postpartum group in New Zealand around 8 years after government interventions. Results will explore whether maternal iodine and selenium status could be used as a proxy measure of infant status. It provides an opportunity to examine the association of maternal iodine and selenium with infant neurodevelopment during their first year. This study explores selenium status using both short-term and long-term measures in relation to neurodevelopment at 6 months and 12 months of age, which has not been reported previously. Furthermore, the study results will add preliminary data on iron status of women at 6 months postpartum.

Importantly, the study will investigate overall thyroid function of women at 6 months postpartum with respect to the risk of postnatal depression. Measurement of thyroid hormones, thyroid stimulating hormone, anti-TPO, Tg, and anti-Tg in serum as well as measuring thyroid gland volume via ultrasound will provide an overall picture of maternal thyroid function after giving birth. This is an opportune time to check thyroid status, especially as women with limited thyroidal reserve or iodine deficiency in pregnancy may develop postpartum thyroid dysfunction, one of the most common endocrine disorders women experience [6,58].

Additionally, there will be longitudinal assessment of mothers’ mental health via repeated screening by using the EPDS. The results may add to the literature in postpartum mental health status. Their offsprings’ growth and neurodevelopment will be followed during the first year after birth. The findings from this study have the potential to inform future public health policy and practice regarding postpartum women’s nutritional status and mental health together with infant health outcomes.

## Appendix

Multimedia Appendix 1 Flow chart showing stages of Mother and Infant Nutrition Investigation study.

## Abbreviations

Anti-Tg: antithyroglobulin antibodies
Anti-TPO: antithyroid peroxidase
ASQ: Ages and Stages Questionnaire
CMIA: chemiluminescent microparticle immunoassay
EPDS: Edinburgh Postnatal Depression Scale
ICP-MS: inductively coupled plasma mass spectrometry
MINI: Mother and Infant Nutrition Investigation
T3: triiodothyronine
T4: thyroxine
Tg: thyroglobulin
