# Supplementary figures and images for: Mother and Infant Nutrition Investigation in New Zealand (MINI Project): Protocol for an Observational Longitudinal Cohort Study
Source: JMIR Res Protoc. 2020 Aug 27;9(8):e18560. doi: 10.2196/18560 (PMC7484772; doi:10.2196/18560)

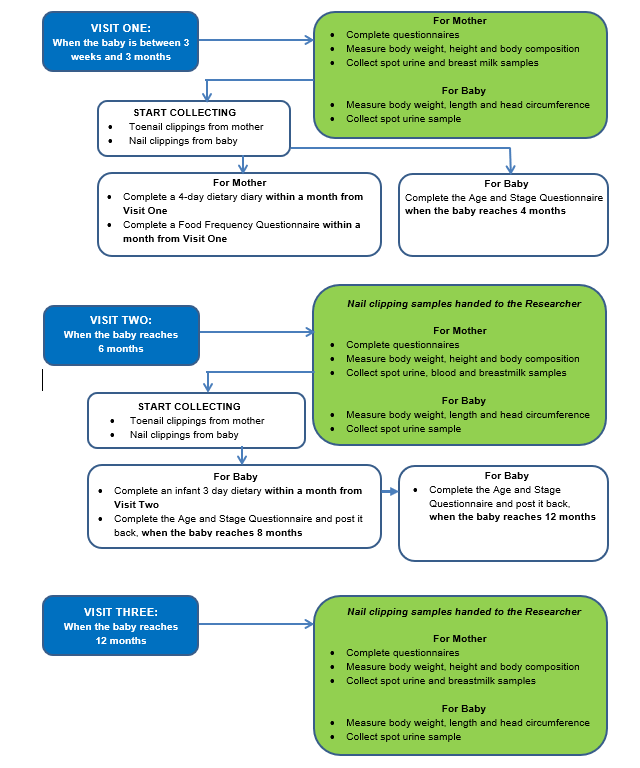

Supplement: Multimedia Appendix 1 [file resprot_v9i8e18560_app1.PNG]
